# Association of HMGB1, IL-1β, IL-8, IL-10, and MCP-1 with the Development of Systemic Inflammatory Response Syndrome in Pediatric Patients with Recently Diagnosed Acute Lymphoblastic Leukemia

**DOI:** 10.3390/life15081187

**Published:** 2025-07-25

**Authors:** Carmen Maldonado-Bernal, Horacio Márquez-González, Erandi Pérez-Figueroa, Rocío Nieto-Meneses, Víctor Olivar-López, Aurora Medina-Sanson, Elva Jiménez-Hernández

**Affiliations:** 1 Unidad de Investigación en Inmunología y Proteómica, Hospital Infantil de México Federico Gómez, Mexico City 06720, Mexico; gera.pfi3@gmail.com (E.P.-F.); qbprocionieto@gmail.com (R.N.-M.); 2 Subdirección de Investigación Biomédica, Hospital Infantil de México Federico Gómez, Mexico City 06720, Mexico; horaciohimfg@gmail.com; 3 Departamento de Microbiología y Parasitología, Facultad de Medicina, Universidad Nacional Autónoma de México, Mexico City 04510, Mexico; 4 Dirección Médica, Hospital Infantil de México Federico Gómez, Mexico City 06720, Mexico; vol63@hotmail.com; 5 Departamento de Hemato-Oncología, Hospital Infantil de México Federico Gómez, Mexico City 06720, Mexico; auroramedina@aol.com.mx; 6 Departamento de Hematología, Hospital Pediátrico Moctezuma, Mexico City 15530, Mexico; elvajimenez@yahoo.com

**Keywords:** acute lymphoblastic leukemia, systemic inflammatory response syndrome, fever, S100A8, HMGB-1, inflammatory cytokines

## Abstract

In acute lymphoblastic leukemia (ALL), neutropenia and fever of unknown origin may occur, indicating the use of antimicrobials to control a probable infection. However, in 60–70% of cases there is no obvious infectious focus so treatment is empirical, increasing the risk of developing systemic inflammatory response syndrome (SIRS). The construction of a prognostic model of fever and development of SIRS based on the identification of endogenous molecules, called alarmins or damage-associated molecular patterns (DAMPs) and inflammatory cytokines, can help identify children with ALL and fever or SIRS and who do not have an infection. A cohort of 30 children with recently diagnosed ALL and absence of infectious microorganisms before starting the remission induction phase was studied. Two groups were identified: (1) a group with SIRS (fever, tachycardia, tachypnea, and leukopenia, without focus of infection) and (2) a group without SIRS. The DAMPs, namely HMGB1 and S100A8 proteins, were quantified by ELISA and inflammatory mediators were determined by multiple protein analysis. The medians of DAMPs and inflammatory mediators in children with SIRS were higher than in children who did not have SIRS, and the delta values of the biomarkers studied in patients with and without SIRS showed important differences, with statistically higher medians in patients with SIRS compared to those without SIRS. HMGB1 together with IL-1β, IL-8, IL-10, and MCP-1 can serve as biomarkers to identify children with ALL and fever or SIRS who should not receive antimicrobial treatment because the origin of their fever is not due to an infectious agent.

## 1. Introduction

Acute lymphoblastic leukemia (ALL) is a mono/oligoclonal cytogenetic alteration that causes the abnormal proliferation of precursor cells of the lymphoid series. It is the most common malignant neoplasm in pediatric age groups, with an incidence of 73 patients per million inhabitants per year, being more common in boys under 5 years of age than in girls [1].

Neutropenia and fever represent the most frequent complications in the treatment of patients with ALL [2]. In these patients, the use of antibiotics and antifungals to control the infection is indicated. However, in up to 60–70% of cases there is no obvious infectious focus, so treatment is empirical. The morbidity of these patients is high, the cost of treatment is high, and their quality of life is reduced. In addition, the application of chemotherapy is delayed.

Fever in cancer patients is defined as an axillary temperature higher than or equal to 38.3 °C in a single reading or an increase in body temperature above 38 °C for a period greater than or equal to one hour [3] The development of fever is a complex phenomenon, mediated by the release of proinflammatory mediators, mainly interleukin-1β (IL-1β), tumor necrosis factor alpha (TNF-α), and IL-6. These cytokines, in addition to inducing fever and inflammatory response, share other properties such as activating T and B cells [4]. They stimulate the production of other inflammatory mediators, which in turn induce lymphocyte activation and monocyte migration, and activate the functions of mature neutrophils, such as chemotaxis, phagocytosis, and bacterial destruction. In the same way, IL-6 induces hepatic synthesis of acute phase proteins, and IL-1β and TNF-α participate in systemic inflammatory response syndrome and multiple organ failure [5]. In a previous study performed by our work group, we found that in pediatric patients with ALL and fever without apparent infection, the TNF-α and IL-6 proinflammatory mediators were increased as well as the IL-8 and MCP1 chemokines and the immunosuppressive IL-10 cytokine [6].

Systemic inflammatory response syndrome (SIRS) is the systemic immune response induced by non-infectious agents. The host inflammatory response in SIRS can lead to multiple organ dysfunction syndrome (MODS) and, ultimately, death. This response begins with the detection of danger by the pattern recognition receptors (PRRs) in immunocompetent cells and endothelia. The detected damage signals, through specific signaling pathways, activate the *κ*B nuclear transcription factor (NF-*κ*B) and other transcription factors and gene regulatory systems that upregulate the expression of proinflammatory mediators, such as cytokines and chemokines. Coagulation cascades are also activated and the acute inflammatory response determines the pathophysiological mechanisms in the development of MODS. Inflammatory mediators affect organ function, directly and remotely, through the production of nitric oxide, which leads to mitochondrial energy and cytopathic hypoxia [5].

It has recently been described that the inflammation that leads to SIRS and multiple organ failure may be mediated by host factors, which include “alarmins”, which are all the products released during necrosis and cell destruction. All of them are called damage-associated molecular patterns (DAMPs) or endogenous ligands of pattern recognition receptors (PRRs) [5]. Endogenous molecules, called “alarmins” or DAMPs, are produced in stressed or damaged tissues or in conditions of altered hemostasis, such as in cancer; however, these molecules have been little studied in cancer [7].

Within the alarmins or DAMPs, the following molecules can be found: HMGB-1 (high-mobility group box-1 protein), HSPs (heat shock proteins), S100 proteins, monosodium urate crystals, uric acid, and ATP. In cancer, alarmins or DAMPs can be released into the surrounding microenvironment during the process of necrosis or immunogenic apoptosis, actively promoting tumor growth or mediating the anti-tumor immune response. The S100 and HMGB1 proteins are among the most studied alarmins related to solid tumors [7].

S100 proteins are a family of small molecular proteins bound to calcium. They usually act as intracellular regulators and as extracellular signaling proteins, and they participate in the processes of differentiation, apoptosis, Ca2+ homeostasis, energy metabolism, inflammation, migration, and invasion. As DAMPs, S100 proteins have been observed to regulate cell proliferation, differentiation, survival, and migration, acting in an autocrine and paracrine manner [8]. The secretion of S100 proteins in the tumor microenvironment facilitates the identification of tumor cells by dendritic cells, increasing immunogenicity. However, the interaction between S100A9 and TLR4 promotes an increase in the production of TGF-β, stimulating tumor growth [9,10].

On the other hand, HMGB1 (high-mobility group box-1) belongs to the superfamily of DNA-binding proteins; it is a non-histone protein that is expressed in all nucleated cells. Under normal conditions, HMGB1 is located in the nucleus and cytoplasm, and depending on its location, the physiological functions that have been described occur [11]. It has been observed that HMGB1 is released into the extracellular environment by different tumors and its interaction with TLR4, TLR9, or RAGE favors the growth and progression of tumors [7]. It has been shown that cancer cells undergoing apoptosis and autophagy also contribute to the release of HMGB1 through the action of 3/7 and ATG5 caspases [11].

In diseases such as juvenile rheumatoid arthritis and gout, uric acid has been shown to play an important role in triggering fever of unknown origin due to hyperuricemia [12]. In the case of patients with ALL, there may be tumor lysis and, with it, an increase in uric acid; however, it has not been associated with the generation of fever or SIRS.

Given the importance of DAMPs in the production of inflammatory mediators, we recently carried out a study in which the general objective was to identify the DAMPs involved in the development of neutropenia and fever in children with ALL without an identified infectious focus. The obtained results showed that the patients had elevated DAMPs, S100A8, HMGB-1 proteins, and uric acid at the time of diagnosis; this implies a possible participation in the inflammatory process and in the generation of fever [6]. Therefore, the aim of the present study was to identify the association of DAMPs, such as HMGB1 and S100A8, and inflammatory mediators, such as TNF-α, IL-8, IL-10, IL-1β, IL-6, and MCP1, in the development of systemic inflammatory response in pediatric patients with recently diagnosed ALL.

## 2. Materials and Methods

A cohort study was carried out in a pediatric hospital in Mexico City from December 2019 to December 2020. Patients aged 1 to 18 years who debuted with ALL were included and were followed during the diagnosis phase, with sample collection, blood tests upon admission, and 7 days later, confirmation that there was no infection at the time of sample collection. Those with infection proven by cultures (blood cultures, urine cultures, stool cultures), procalcitonin greater than 0.5 pg/dL, administration of antimicrobials thirty days before diagnosis and during the follow-up time, indication of antipyretics 6 h before taking laboratory tests, initiation of chemotherapy before day 7, and whose ALL was their second neoplasm were excluded. All patients included in the study were treated with standard clinical practices.

To enter the protocol, all parents of the patients signed a letter of informed consent, and the threshold age of the patients for providing assent was 8 years old. The manuscript was approved by the Ethics, Research, and Biosafety Committees from the Federico Gómez Children’s Hospital of Mexico in Mexico City with registration number HIM/2018/077 SSA1528.

Cohort time zero was defined during the first 24 h of hospitalization when baseline samples were taken. Follow-up was carried out for 7 days, with a new sample taken to determine inflammatory mediators.

The outcome of interest was the development of SIRS during the first 7 days, defined as leukopenia, in addition to an axillary temperature higher than or equal to 38.3 °C in a single reading or an increase in body temperature above 38 °C for a period greater than or equal to one hour, or a temperature less than 36 °C, tachypnea, or tachycardia. Other secondary outcomes were death or the presence of sepsis.

The demographic variables recorded were sex, age at diagnosis, anthropometry, previous comorbidities, and syndromic alterations.

The variables of interest were the DAMPs, HMGB1 and S100A8, and the TNF-α, IL-8, IL-10, IL-1β, IL-6, and MCP1 serum inflammatory mediators. DAMPS (HMGB1 and S100A8) were quantified by ELISA. Briefly, polystyrene microplates were sensitized with a specific monoclonal antibody against each of the molecules of interest, anti-HMGB1 (Cat. MBS701378), and anti-S100A8 (Cat. MBS2504193), from MyBiosourse, San Diego, California, United States. A total of 100 µL of the patients’ sera was added, along with the dilutions of the standard of the corresponding molecule, and were incubated for 2 h at room temperature; studies were performed in duplicate. A second antibody specific for each molecule, labeled with biotin and an enzymatic agent conjugated with HRP-avidin, was added and the TMB substrate solution (3,3′,5,5′ tetramethylbenzidine) was added to reveal the enzymatic reaction; the procedure was performed according to the manufacturer’s instructions. Between each step, washes were carried out with a pH 7.2 phosphate buffer solution. The absorbance was measured at 450 nm in the spectrometer (Thermo Scientific, Multiscan FC, Shanghai, China) and the results were expressed in pg/mL based on the standard curves for each test molecule. Inflammatory mediators were quantified by multiple protein analysis (xMAP). Briefly, 50 µL of patient sera was used, in duplicate, and beads labeled with antibodies specific for each inflammatory mediator. MILLIPLEX MAP Human Cytokine/Chemokine Magnetic Bead kits were used (Millipore Cat. HCYTOMAG-60K, Burlington, MA, USA), which contain microspheres labeled with a specific capture antibody, which binds to the molecule of interest, to subsequently be detected through a molecule reporter labeled with phycoerythrin. Using high-speed digital signal processors, the microspheres were identified individually and the results of the assay, based on fluorescence signals, were quantified with a Luminex MAGPIX^®^ device (Milliplex^®^; EMD Millipore, Austin, TX, USA). The procedure was performed according to the manufacturer’s instructions and the results were expressed in pg/mL. The statistical analysis method was the Wilcoxon matched-pairs signed rank test.

The delta value (Delta Sign) was calculated between the baseline measurement and day 7 with the following formula:

*∆* = *baseline cytokine or DAMP value* − *day 7*, *cytokine or DAMP value*/*baseline cytokine or DAMP value*.

The type of leukemia, the leukemia risk classification, the need for hospitalization in an intensive care area, the development of tumor lysis syndrome, the need for transfusion of any type of blood products, and the beginning of the steroid window were considered as confounding variables.

A sample size calculation was performed for a non-finite population based on the previous study, calculating the probability of presenting a SIRS outcome, with a result of 30 patients.

Statistical analysis: Normality tests, namely the Shapiro–Wilk test, demonstrated a non-parametric distribution of the data, which were expressed in medians and interquartile ranges. Qualitative variables were represented in absolute numbers and percentages. The incidence and cumulative incidence of SIRS were calculated.

For the inferential analysis, patients with and without SIRS were established as comparison groups and χ^2^ tests were used for qualitative variables and the Mann–Whitney U test for quantitative variables. The statistical program used was SPSS. Version 25 for MAC IBM.

## 3. Results

The results shown are from 18 patients recently diagnosed with ALL, with the Pre B immunophenotype, 7 girls and 11 boys, with an average age of 5.3 years (range of age 3.8–8.8); all were free of infection during the 7 days of observation and monitoring. Two groups were identified, a group of 14 children who did not develop SIRS and a group of 4 children who developed SIRS (fever, tachycardia, tachypnea, and leukopenia, and with no source of infection) (Table 1). Twelve patients who did not meet the study criteria were excluded.

The median age of the children who did not have SIRS was 6 years and that of the children who had SIRS was 4.6 years; the difference was significant, *p* < 0.05.

In the same way, at the time of diagnosis, the median leukocyte count of patients without SIRS was higher than that of patients with SIRS, *p* < 0.05. The median lymphocyte count, neutrophils, and eosinophils were higher in the group of children without SIRS compared to children with SIRS, but the differences were not significant (Table 2). The higher proportion of leukocytes found in patients without SIRS is likely due to the fact that there is a higher proportion of neutrophils in these patients than in those with SIRS, *p* = 0.05. The comparisons in Table 2 show the median of the group with SIRS versus the median of the group without SIRS, with an interquartile range of p25 and p75.

**Figure 1:**
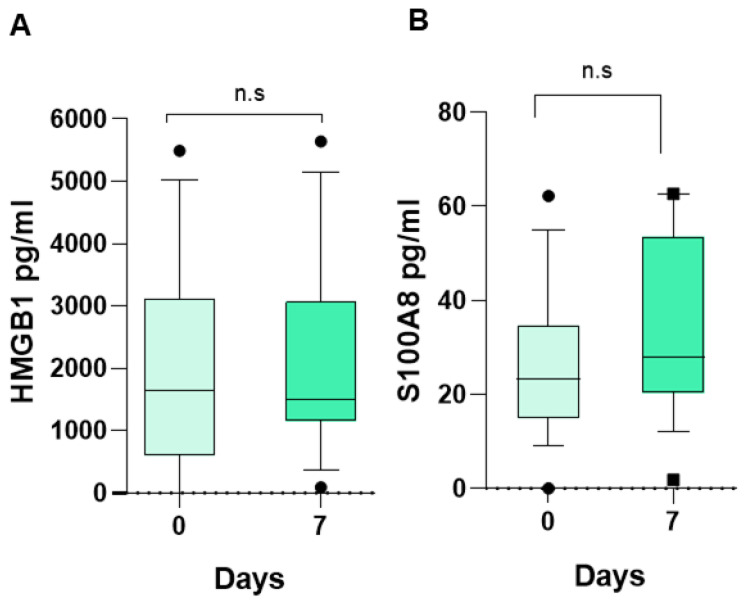
Damage-associated molecular patterns (DAMPs) in children with acute lymphoblastic leukemia (ALL). Median serum concentrations of HMGB1 (**A**) and S100A8 (**B**) of patients with ALL at admission (0 days) and 7 days later. Wilcoxon matched-pairs signed rank test. ns, not significant.

In relation to DAMPs or alarmins, we observed that the median of HMGB1 at the time of diagnosis was 1647 pg/mL (0.0–5494 pg/mL) (Figure 1A) and that of S100A8 proteins was 23.3 pg/mL (0–62.9 pg/mL) (Figure 1B), and at 7 days of follow-up, the mean HMGB1 was 1509 pg/mL (94.0–5644 pg/mL); it did not change significantly, as expected, and S100A8 proteins did not change either, being 27.9 pg/mL (1.82–62.73 pg/mL) (Figure 1A,B). This suggests that the patients remained free of any infectious process during these 7 days.

**Figure 2:**
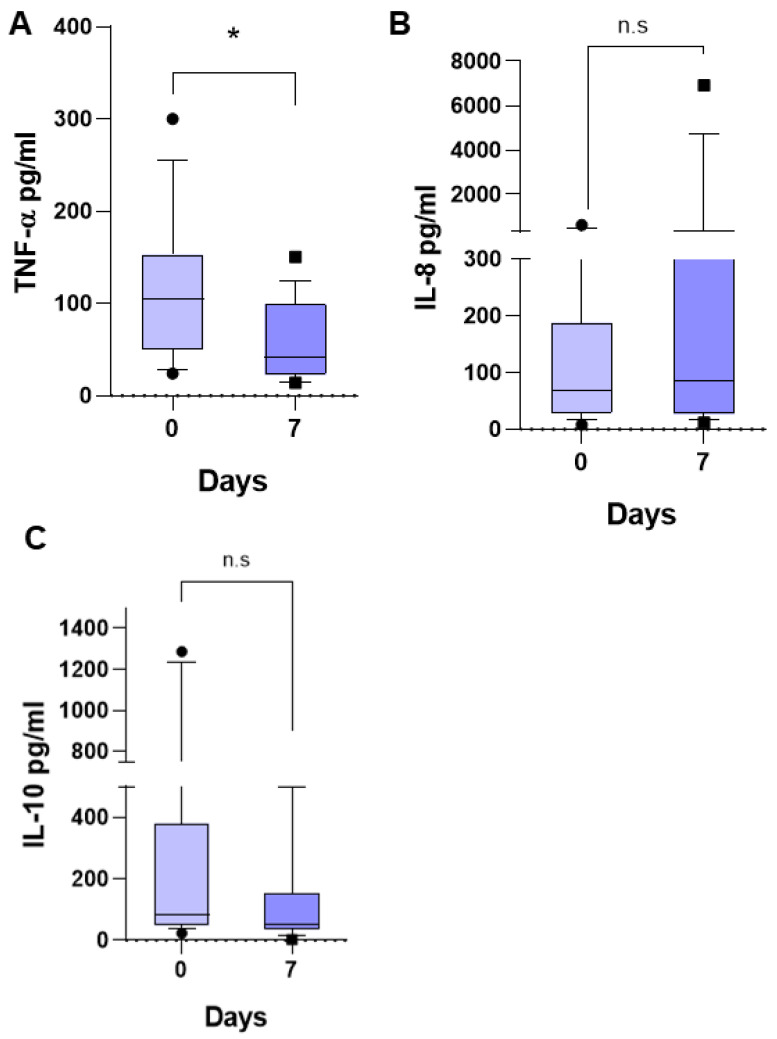
Inflammatory cytokines in children with acute lymphoblastic leukemia (ALL). Median serum concentrations of TNF-α (**A**), IL-8 (**B**), and IL-10 (**C**) at admission (0 days) and 7 days later of patients with ALL. Significant difference Wilcoxon matched-pairs signed rank test, * *p* < 0.05. ns, not significant.

Regarding the three inflammatory mediators previously studied and found to be associated with fever in pediatric patients with ALL (6) in the present study, we observed that the median TNF-α was of 104.7 pg/mL (24.31–303.3 pg/mL) (Figure 2A), that of IL-8 was 69.19 pg/mL (8.23–623.2 pg/mL) (Figure 2B), and that of IL-10 was 88.58 pg/mL (37.83–1287 pg/mL) (Figure 2C) at the time of diagnosis. At 7 days of follow-up, the TNF-α median decreased to 42.21 pg/mL (14.06–150.4 pg/mL), *p* < 0.0044, that of IL-8 had no significant change, being 85.96 pg/mL (11.48–6910 pg/mL), and that of IL-10 decreased to 53.87 pg/mL (3.2–510.9 pg/mL), but without a significant difference, *p* = 0.2734. This suggests that the patients remained free of any infectious process during these 7 days, and the significant reduction in TNF-α at 7 days of follow-up suggests that the care conditions in the hospital favored the patients, reducing the inflammatory response.

**Figure 3:**
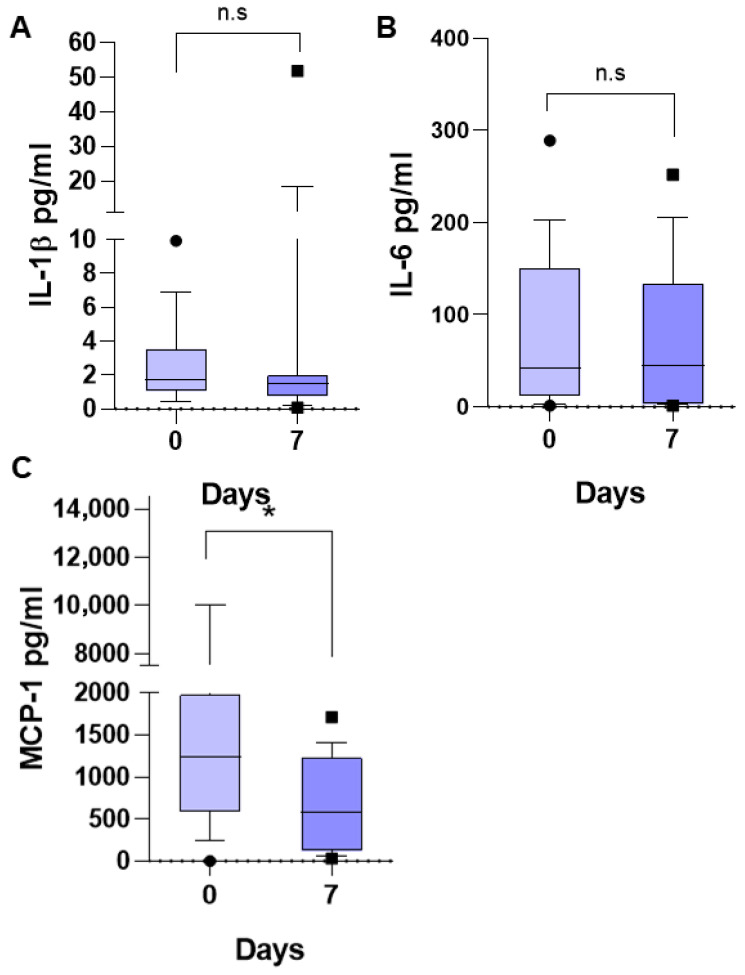
Inflammatory cytokines and MCP-1 in children with acute lymphoblastic leukemia (ALL). Median serum concentrations of IL-1β (**A**), IL-6 (**B**), and MCP-1 (**C**) at admission (0 days) and 7 days later of patients with ALL. Wilcoxon matched-pairs signed rank test, * *p* < 0.05. ns, not significant.

The medians of the other three determined inflammatory mediators did not undergo a significant change at 7 days of follow-up with respect to the initial values. IL-1β at the time of diagnosis was 1.07 pg/mL (0.41–9.9) (Figure 3A), IL-6 was 41.72 pg/mL (1.39–289.9 pg/mL) (Figure 3B), and MCP-1 was 1239 pg/mL (0.0–10,000 pg/mL) (Figure 3C). At 7 days of follow-up, the median IL-1β was 1.5 pg/mL (0.09–51.67), that of IL-6 was 44.8 pg/mL (1.5–252.2), and that of MCP-1 was 575 pg/mL (32.25–1711), * *p* < 0.05.

**Figure 4:**
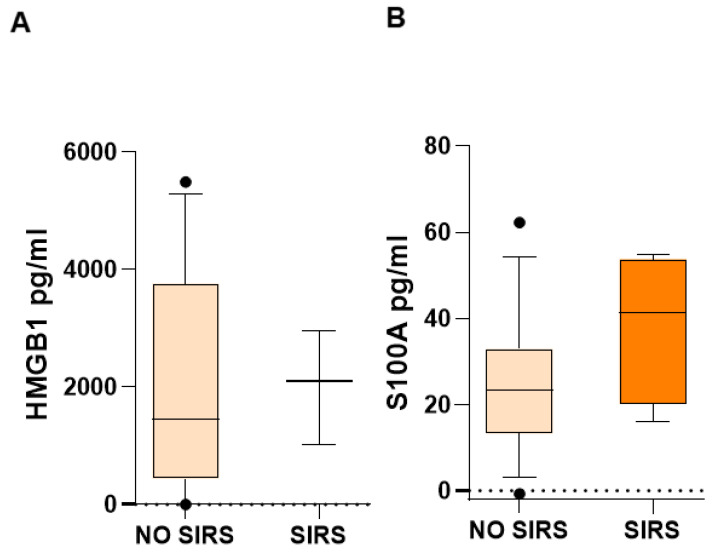
Biomarker concentrations in patients with and without SIRS in children with acute lymphoblastic leukemia. Important differences were found in median serum concentrations of HMGB1 (**A**) and S100A8 (**B**). U Mann–Whitney test.

The medians of DAMPs and inflammatory mediators in children with SIRS were higher than in children who did not have SIRS: HMGB-1, 2099 vs. 1459 pg/mL (Figure 4A); S100A8, 41.28 vs. 23.30 pg/mL (Figure 4B). However, probably due to the dispersion of the data and the small number of patients, the difference was not significant, but the increase is very evident.

**Figure 5:**
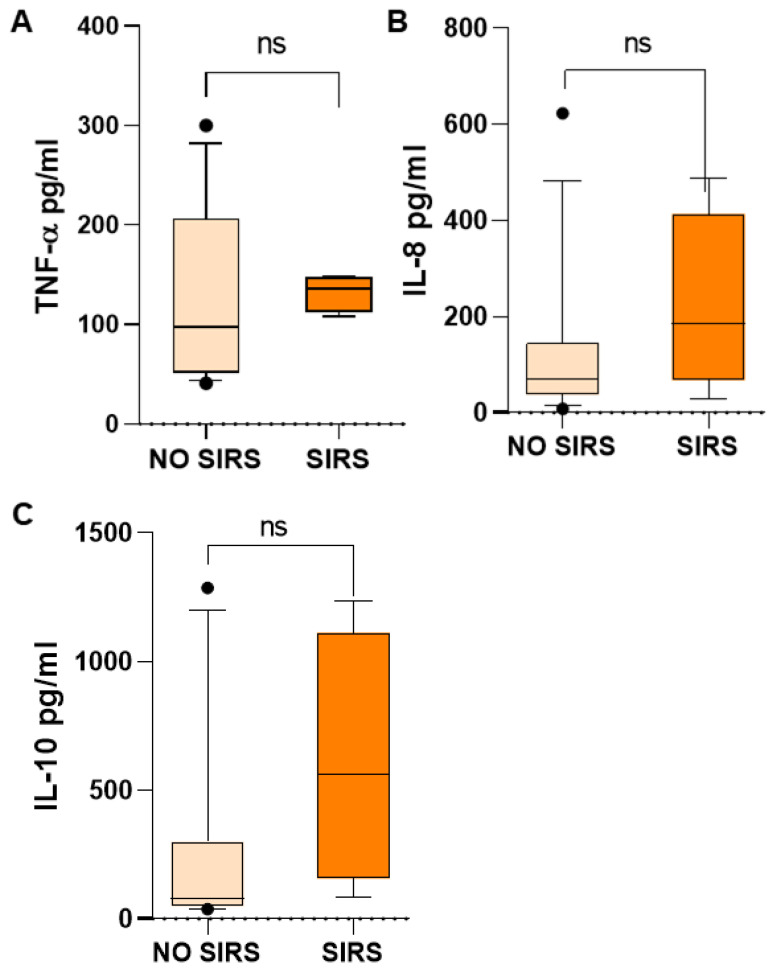
Biomarker concentrations in patients with and without SIRS in children with acute lymphoblastic leukemia. Important differences were found in median serum concentrations of inflammatory cytokines TNF-α (**A**), IL-8 (**B**), and IL-10 (**C**). U Mann–Whitney test. ns, not significant.

Inflammatory mediators in children with SIRS were higher than in children who did not have SIRS, such as TNF-α, 135.73 vs. 97.52 (Figure 5A); IL-8, 185.73 vs. 69.19 (Figure 5B); and IL-10, 564.27 vs. 81.21 pg/mL (Figure 5C). However, probably due to the wide dispersion of the data, mainly in the group of patients without SIRS, the difference was not significant, but the increase is very evident. TNF-α and IL-8 are proinflammatory mediators and IL-10 is an anti-inflammatory mediator, which is very high in patients with SIRS, without being able to counteract the inflammatory response in these patients.

**Figure 6:**
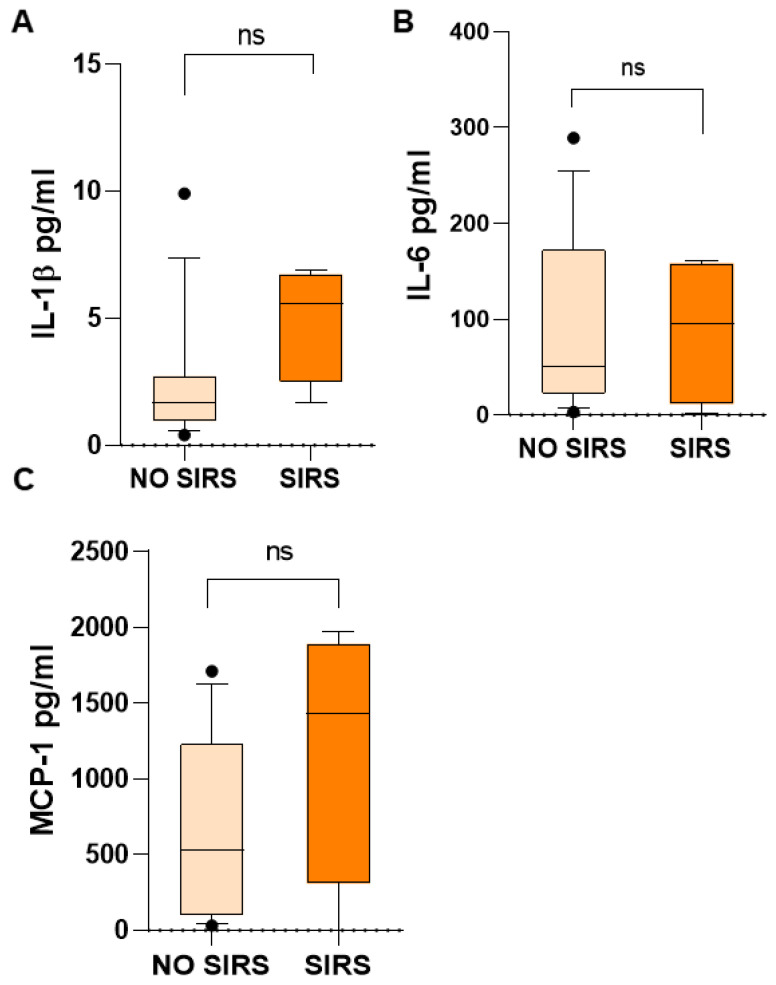
Biomarker concentrations in patients with and without SIRS in children with acute lymphoblastic leukemia. Important differences were found in median serum concentrations of inflammatory cytokines IL-1β (**A**), IL-6 (**B**), and MCP-1 (**C**). U Mann–Whitney test. ns, not significant.

Additionally, IL-1β, IL-6, and MCP-1 in children with SIRS were higher than in children who did not have SIRS: IL-1β, 5.57 vs. 1.69 (Figure 6A); IL-6, 95.89 vs. 50.35 (Figure 6B); MCP-1, 1434 vs. 531.4 (Figure 6C) pg/mL. However, probably due to the wide dispersion of the data and the small number of patients, the difference was not significant, but the increase is evident. All of them are proinflammatory mediators.

**Figure 7:**
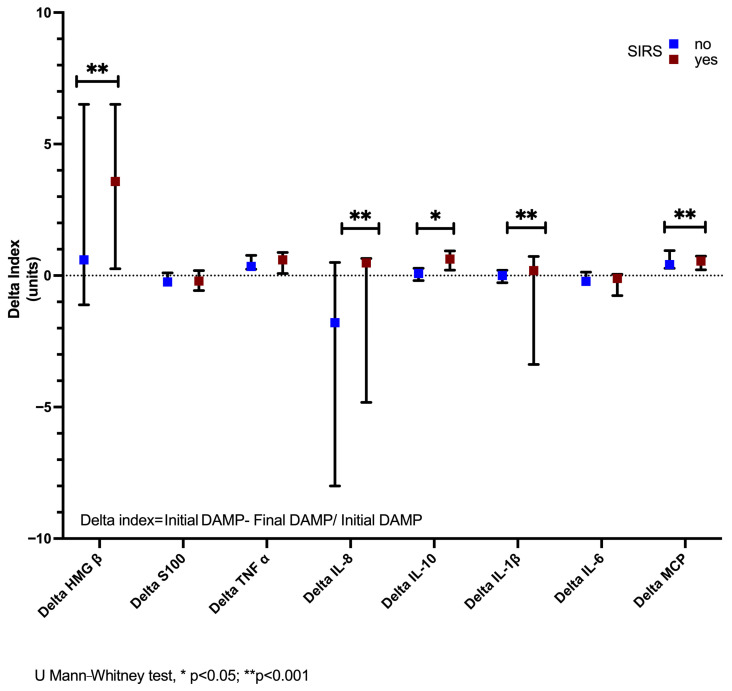
Delta values of the biomarkers in patients with and without SIRS. Important differences were found in HMGB1, IL-8, IL-10, and IL-1β, with statistically higher medians in patients with SIRS than those without SIRS. U Mann–Whitney test, * *p* < 0.05, ** *p* < 0.001.

In relation to the delta values of the biomarkers in patients with and without SIRS, important differences were found, with statistically higher medians in patients with SIRS compared to those without SIRS of ∆ HMGB1 [3.58 (0.26–6.5) vs. 0.6 (−1.1–6.5)], ∆ IL-8 [0.3 (−4–0.05) vs. −2 (−8–0.05)], ∆ IL-10 [0.63 (0.21–0.94) vs. 0.08 (−0.19–0.28)], ∆ IL-1β [0.19 (−3.4–0.73 vs. −1.79 (−8–0.1)], and ∆ MCP1 [0.55 (0.22–0.74) vs. 0.42 (0.28–0.95)], * *p* < 0.05 (Figure 7).

## 4. Discussion

Fever in children with acute lymphoblastic leukemia frequently does not respond to the use of antimicrobials. This is because the treatment is empirical because the infectious microorganism cannot be isolated and the systemic inflammatory response syndrome (SIRS) can be triggered, which is often confused with sepsis. In a previous work we demonstrated that children who present febrile symptoms without apparent infection have elevated concentrations of HMGB1 and S100A8 proteins (unpublished data). Therefore, in this work, we researched whether elevated concentrations of HMGB1 and S100A8 in children with acute lymphoblastic leukemia are associated with the presentation of systemic inflammatory response syndrome.

The difference in the therapeutic approach implies the differentiation of SIRS and non-SIRS in children with ALL without apparent clinical infection. Here, we show that elevated concentrations of HMGB1 have the potential to make such a distinction in a group of patients with acute lymphoblastic leukemia, without apparent clinical infection.

In our study, the demographic characteristics and ALL severity were similar in patients who developed SIRS and patients who did not develop SIRS, except for the mean age of children with SIRS, which was younger than that of children without SIRS.

Children with SIRS had a lower number of leukocytes than children without SIRS, which may predispose them to a greater risk of infections. However, and as expected, during the 7 days of observation, they did not present any infection. The reduction found in leukocytes in children with SIRS cannot be attributed to the age difference between the two groups, since it is known that younger children have a higher number of leukocytes, as was the case in this group.

We observed that the concentration of HMGB1 at 7 days of follow-up had not increased in the group of 18 included children, which suggests that there was no infectious process; this was confirmed by several negative cultures. Similarly, the S100A8 protein at 7 days of follow-up had not increased.

The inflammatory cytokines associated with fever in patients with ALL (IL-8 and IL-10) [6], as expected, did not increase after 7 days of follow-up, suggesting that there was no infectious process and that it may be a process of sterile inflammation, such as that which occurs with other pathologies such as rheumatoid arthritis. However, TNF-α showed a significant reduction at 7 days of follow-up, which suggests that the care conditions in the hospital favored the patients, reducing the inflammatory response. A strong and uncontrolled inflammatory response is a characteristic of patients with ALL [6].

Regarding the inflammatory cytokines IL-1β and IL-6, as expected, there was no change at 7 days of follow-up. However, MCP-1 showed a significant reduction at 7 days of follow-up, which again suggests that no infectious process occurred during this time of observation of the patients and that the care conditions in the hospital favored the patients, reducing the inflammatory response.

All of the above allows us to consider that none of the included patients had an infectious process and that we can compare the patients who did not have SIRS with those who developed SIRS and determine their association with the studied DAMPs and inflammatory mediators.

The medians of the DAMPs, HMGB1 and S100, in children with SIRS were higher than in children who did not have SIRS. Also, inflammatory mediators TNF-α, IL-8, IL-10, IL-1β, IL-6, and MCP-1 in children with SIRS were higher than in children who did not have SIRS. But the differences were not significant, probably due to the small number of patients and the wide dispersion of the results.

However, the deltas of the DAMP, HMGB1, in the group that developed SIRS were much higher than those of the group that did not develop SIRS and were above zero, which allows us to infer that it is associated with SIRS in patients with ALL without apparent infection.

HMGB1 has been identified as an important mediator of local and systemic inflammatory diseases when released into the extracellular milieu. It has potent inflammatory effects and acts as a DAMP when released from cells; it can trigger massive production of inflammatory cytokines from immune response cells through different signaling pathways, involving pattern recognition receptors such as TLR4 and RAGE [13,14,15]. The fact that it is associated with SIRS in patients with ALL and without apparent infection allows us to propose it as a marker to identify patients who will develop a strong inflammatory response in the absence of infectious microorganisms. This is of great importance for it allows us to indicate that antimicrobials should not be administered, since they are not required and their use would only delay antineoplastic treatment, which could generate comorbidities and longer hospital stays. HMGB1 has been proposed as a useful molecule to differentiate patients with SIRS and sepsis in pathologies such as secondary peritonitis [16]. In addition, HMGB1 is an important target in inflammatory processes; it has been shown in models of ischemia reperfusion injury (IRI), e.g., renal IRI, that the administration of an anti-HMGB1 antibody reduces inflammation. Additionally, this latter study demonstrated that TLR4^−/−^ mice are protected from renal IRI and are insensitive to the effects of HMGB1 manipulation [17,18]. However, until now, its usefulness has not been seen as a marker for patients with ALL who have SIRS and who should not receive antimicrobial treatment as their inflammatory response is due to the activation by DAMPs and not to infectious microorganisms. In 2007 it was reported [11] that HMGB1 was found to be increased in children with ALL compared to healthy children, and was reduced when children were in remission.

S100A8 is a protein expressed mainly in neutrophils and monocytes and is known to play a critical role in modulating inflammatory responses [19]. In this study, we found it elevated in patients with SIRS compared with those who did not present SIRS. However, the difference was not significant. The presence of S100A8 may affect platelet function and, therefore, generate thrombosis. S100A8/A9 induced phosphatidylserine (PS) exposure but failed to induce platelet aggregation, despite GPIIb/IIIa activation and alpha-granule secretion [20]. Also, S100A8/A9 could induce neutrophil activation, including adhesion and CD11b upregulation, indicating that this DAMP might amplify neutrophil activation. It is a marker for the release of Neutrophil Extracellular Traps (NETS) and induces neutrophil activation [21].

In this study, HMGB1 was found to be associated with SIRS in children with ALL without apparent clinical infection. In the same way, the IL-8, IL-10, IL1-β, and MCP-1 inflammatory mediators had higher deltas (above 1) in the group with SIRS than in the group without SIRS, which suggests that they are associated with the development of SIRS. IL-8 is a chemokine that recruits leukocytes, mainly neutrophils, with which there may be a greater release of inflammatory mediators; IL-10 can inhibit the production of IL-12 and costimulatory and MHC class II molecules of macrophages and of dendritic cells, acting as an immunoregulatory and immunosuppressive cytokine; however, we observed that it was deregulated and associated to fever in the previous study [6]. On the other hand, it is known that IL-1β can produce fever by acting on the hypothalamus, in addition to inflammation and coagulation when it acts on endothelial cells; at the liver level, it induces the synthesis of acute phase proteins, which may affect patients with SIRS. Also, the MCP-1 delta was higher in the group with SIRS compared to the group without SIRS, which implies that in patients with SIRS there may be more recruitment of lymphocytes than in patients without SIRS.

The TNF-α and IL-6 cytokines were not associated with SIRS; the deltas of the two groups were above zero.

This study was well controlled, both at baseline and during follow-up, and the included patients met all inclusion criteria. However, the main limitation of this study is that there were few patients. Nonetheless, we have the previous study that included 99 patients [6] and unpublished data, which allows us to be confident in what was observed in this study.

The advantages of this study consist of the selection criteria (patients without chemotherapy and without initial infectious focus and confirmed during follow-up) that allowed us to identify the association of HMGB1, IL-8, IL-10, IL1-β, and MCP-1 with leukemic activity and the generation of SIRS. This same situation, with such strict criteria, conditioned a small sample size.

The novelty of this work is the association of DAMPs (HMGB1) and inflammatory mediators (IL-8, IL-10, IL1-β, and MCP-1) in the development of systemic inflammatory response (SIRS) in pediatric patients with newly diagnosed acute lymphoblastic leukemia (ALL) and no apparent clinical infection. There is information in the literature about DAMPs [5,7,8] and inflammatory mediators [4,5,6] and their participation in different cancers [9,10,11], but no work has reported their association in the development of SIRS in ALL.

## 5. Conclusions

These results may lay the foundations for identifying the group of patients with SIRS not associated with microorganisms and, therefore, avoid the habitual use of empirical antimicrobials, which will not solve the problem of fever and will prolong treatment, increasing the risk of comorbidities. In patients with ALL and fever or SIRS, the application of prognostic models that anticipate the evolution to SIRS may make it possible to reduce the indication for antimicrobials.

## Figures and Tables

**Table 1:** Characteristics of the patients.

Characteristic	Median (p25–p75)	%
Sex		
Girl	7	38.9
Boy	11	61.1
Diagnostic		
ALL1	13	72.2
ALL2	5	27.8
Immunophenotype Pre B		100.0
Age (years)	5.3 (3.8–8.8)	
Weight (kg)	17.2 (14–43)	
Height (cm)	108 (99–129)	
NO SIRS	14	77.8
SIRS	4	22.2

**Table 2:** Anthropometric and initial blood count differences between patients with leukemia with and without SIRS.

	Without SIRS			With SIRS			
	14			4			
Characteristics	Median	p25	p75	Median	p25	p75	*p*
Age (years)	6.0	3.8	12.0	4.6	3.15	5.6	<0.05
Weight (kg)	17.25	14.5	44.0	17.6	15.1	19.5	0.9
Height (cm)	106	69	162	110	104	129	0.8
Leukocytes (cel/mm^3^)	24,050	14,400	58,200	4235	2285	26,680	<0.05
Lymphocytes (cel/mm^3^)	71.5	50.0	89.3	64.5	33.5	203.5	0.07
Neutrophils (cel/mm^3^)	1858.0	712.84.0	2425.0	1010.5	535.9	1335.5	0.05
Eosinophils (cel/mm^3^)	0.03	0.00	0.38	0.00	0.00	0.10	0.8
Creatinine (mg/dL)	0.45	0.40	0.59	0.46	0.03	0.63	0.8

## Data Availability

The datasets used and/or analyzed during the current study are available from the corresponding author on reasonable request.
